# The Bactericidal Activity and Spore Inhibition Effect of Manuka Honey against *Clostridioides Difficile*

**DOI:** 10.3390/antibiotics9100684

**Published:** 2020-10-09

**Authors:** Lillian Yu, Reynal Palafox-Rosas, Brian Luna, Rosemary C. She

**Affiliations:** 1 Department of Pathology, Keck School of Medicine of the University of Southern California, Los Angeles, CA 90033, USA; lilliy1@uci.edu (L.Y.); palafoxr@usc.edu (R.P.-R.); 2 Department of Molecular Microbiology and Immunology, Keck School of Medicine of the University of Southern California, Los Angeles, CA 90033, USA; brianlun@usc.edu

**Keywords:** methylglyoxal, spore, *C. difficile* infection

## Abstract

*Clostridioides difficile* colitis overgrowth occurs when the normal gut microbiome becomes disrupted, often due to antibiotics. Effective treatment remains elusive, due partly to the persistence of its spores in the gut. Natural substances like manuka honey offer an alternative antimicrobial mechanism of action to conventional antibiotics. We investigated the antibiotic activity of manuka honey against 20 *C. difficile* isolates. The minimum inhibitory concentrations (MICs) and minimal bactericidal concentrations (MBC) of manuka honeys of methylglyoxal (MGO) grades 30^+^, 100^+^, 250^+^, and 400^+^ were determined based on broth microdilution. Sporicidal activity was assessed in a range of honey concentrations by enumerating total viable cell and spore counts at 0–96 h after organism inoculation. The MICs of *C. difficile* ranged from 4% to >30% (*w*/*v*). MIC_50_ for the four MGO grades were similar at 10–14%. MBC results for the majority of isolates were distributed bimodally at MBC/MIC ratios ≤4 or MBC >30%. Growth kinetics in honey showed total viable cell counts remaining >10^5^ colony-forming units (CFU)/mL at all time points, whereas spore counts remained within 1-log of baseline (10^2^ CFU/mL) in honey but steadily increased in the drug-free control to >10^5^ CFU/mL by 96 h. Manuka honey demonstrated variable inhibitory and bactericidal activity against *C. difficile*. MGO grade had no noticeable impact on overall MIC distributions or bactericidal activity. Although manuka honey could inhibit spore proliferation, it did not eradicate spores completely.

## 1. Introduction

*Clostridioides difficile* is a major cause of antibiotic-associated diarrhea worldwide, accounting for 20% of cases among hospitalized patients [1]. The incidence of infections varies with geographic region, with a low incidence reported in Asia, whereas, in the U.S., *C. difficile* is the most common pathogen causing healthcare-associated infection [2,3]. This spore-forming Gram-positive bacterium can colonize the gastrointestinal tract and cause a toxin-mediated disease when the gut microbiome is disrupted, most commonly from antibiotic therapy. *C. difficile* infection (CDI) is difficult to eradicate despite antibiotic treatment, with a recurrence rate of 20–25%. Therapeutic failure is generally caused by the survival of organisms in the spore form, persistence in gastrointestinal mucosa biofilm, and host factors that increase vulnerability, rather than by antibiotic resistance [4,5]. Modulation of the host response to prevent CDI or recurrence such as through vaccination remains investigative at this time [6]. Standard therapy for *C. difficile* infection (CDI) includes vancomycin or metronidazole, which, while active against vegetative organisms, do not effectively inhibit spore formation [7]. Investigations of compounds capable of killing spores have expanded to biological compounds such as lauric acid, nisin, lysozyme, and bactericidal peptides [8,9]. Natural products such as cinnamon root powder, peppermint oil, and fresh garlic bulb extract have been found to inhibit growth and metabolic functions in *C. difficle* in vitro [10]. *C. difficle* toxin production and activity may also be reduced by the natural compounds fresh onion bulb extract, zingerone, and Manuka honey [11]. Manuka honey, derived from the Manuka tree (*Leptospermum scoparium*), has also been found to have an antimicrobial effect against *C. difficile* in a handful of other studies [10,12,13]. Manuka honey contains a host of phytochemicals with antimicrobial activity that synergize with the main antibiotic component methylglyoxal (MGO) [14,15,16]. In addition to the antimicrobial activity of manuka honey, its potential benefits include its capacity to promote wound healing and its safety as a dietary supplement [17,18]. Interestingly, a case report demonstrated that administration of manuka honey via endoscopic lavage to a patient with persistent CDI resulted in clinical cure. Despite the positive therapeutic outcome, the authors did not evaluate the in vitro susceptibility of the *C. difficile* isolate to manuka honey [19]. 

Additional studies are needed to assess the antimicrobial activity of manuka honey against clinical *C. difficile* isolates and its effect on *C. difficile* spore counts. Our objective was to further characterize the in vitro antibacterial effect of manuka honey against toxigenic *C. difficile,* including isolates from patients suspected of CDI to better assess the clinical relevance of our findings.

## 2. Results

### 2.1. Minimal Anhibitory and Bactericidal Concentrations

**Figure 1:**
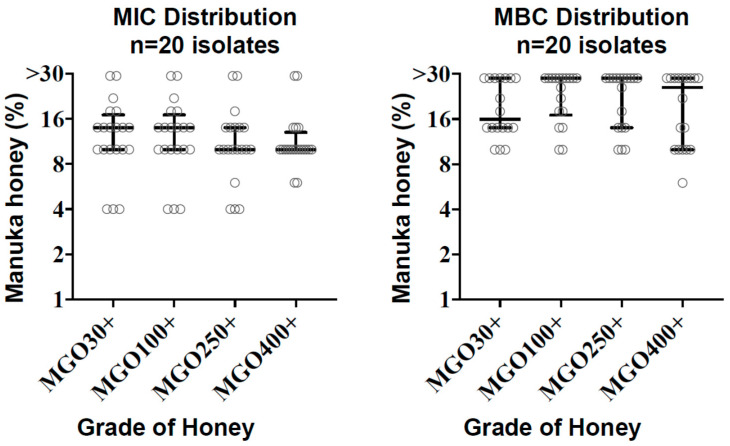
Distribution of minimum inhibitory concentration (MIC) and minimal bactericidal concentrations (MBC) values with median and interquartile ranges are shown for each methylglyoxal (MGO) grade of honey tested against *C. difficile* (n = 20).

The minimum inhibitory concentrations (MICs) of *C. difficile* isolates (n = 20) ranged from 4% to >30%. The MIC_90_ was 22% for MGO 30^+^ and 100^+^ honeys, 18% for MGO 250^+^, and 14% for MGO 400^+^. MIC_50_ values were similar at 10–14% for all honey grades (Table 1, Figure 1). Two isolates yielded MICs of >30% for all honeys tested. There were no significant differences in MIC values between MGO grades (*p* = 0.57, Kruskal–Wallis test). Intra-assay reproducibility based on duplicate MIC testing demonstrated mean coefficients of variation of 4.7–7.1% depending on the MGO grade of honey, and 5.8% across all results.

The observed bactericidal activity was variable depending on the grade of honey and the clinical isolate (Figure 1). Roughly half of the isolates demonstrated some measurable bactericidal activity, in which the minimal bactericidal concentrations (MBC)/MIC ratio was ≤4, whereas bactericidal activity was beyond the upper limit of 30% honey concentration in the remainder of isolates (Table 1, Supplementary Data).

### 2.2. Total Viable and Spore Concentrations in Presence of Manuka Honey

**Figure 2:**
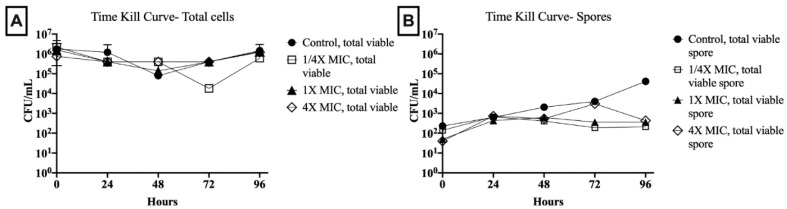
Time-kill studies illustrate the (**A**) total vial cell and (**B**) total viable spore concentrations over 96-h in the presence of varying manuka honey concentrations (1/4×, 1×, and 4× the MIC) and honey-free control. *Abbreviations:* CFU, colony-forming units; MIC, minimal inhibitory concentration.

Total viable cell and spore counts were assessed in 1/4×, 1×, and 4× the MIC of an MGO 400^+^ honey with 6% MIC and 30% MBC against the tested *C. difficile* isolate. Over the 96-h period, under all test conditions, the total viable cell count remained >10^5^ colony-forming units (CFU)/mL, except for sporadic single time point decreases of 1- to 2-log (Figure 2). Spore counts in drug-free control showed a steady increase to >10^4^ CFU/mL by 96 h. In relation to total viable cells, this represented an increase from 0.01% to 1% of the total cell count from baseline to the 96-h endpoint. Spore counts in 1.5% (1/4× MIC) and 6% (1× MIC) honey concentrations remained within 1-log of baseline (10^2^ CFU/mL) throughout the 96-h period. In 24% honey (4× MIC), spore concentrations stayed within 1-log of baseline except for a single increase to 10^3^ CFU/mL at 72 h. However, at 96-h, for all honey concentrations, spore concentrations were <0.04% of total viable cells. 

## 3. Discussion

Because few other studies have examined the antimicrobial effect of manuka honey against *C. difficile,* our aim was to augment the number of clinical *C. difficile* isolates tested against a variety of MGO grades of manuka honey. To date, *C. difficile* has been reported to have uniformly low MICs and MBCs of 6–7% with one strain type and 22 clinical isolates of different strain types [12,13]. Using a standard broth microdilution method, we demonstrated that the activity of manuka honey against *C. difficile* were more variable than seen in prior studies, with MICs ranging from 4% to >30%. Thus, individual strain differences must be taken into account when considering manuka honey as a therapeutic potential against CDI. We further found that MGO grade did not have a significant impact on MIC values and, generally, MICs obtained by different MGO grades agreed within a two-fold difference. This finding is consistent with our previous work, which showed that higher grades of manuka honey did not necessarily confer increased antimicrobial effect against bacterial pathogens by in vitro susceptibility testing [20].

We found the broth microdilution method to have strong reproducibility between replicates and similar trends in MIC results across different MGO grades of honey. Previous studies employed other susceptibility test methods, such as larger volume broth dilution (macrodilution) with or without a standardized organism inoculum. Small differences between MIC values obtained by broth microdilution versus macrodilution methods have been noted with Gram-negative organisms, but either method is an accepted approach for susceptibility testing according to clinical laboratory standards [21]. The lack of standardization for MIC testing of *C. difficile* is evident by the variation seen in research methods and the infrequency of such testing in clinical laboratories, supported by the concept that relapse in *C. difficile* infection is most often due to reasons other than antimicrobial resistance [4]. While broth microdilution method has acceptable agreement with the gold standard agar dilution method for *C. difficile* against a number of conventional antibiotics [22], there remain reproducibility issues for other agents [23]. These considerations are warranted when interpreting the results of experimental studies for testing *C. difficile* with non-conventional agents.

While able to inhibit proliferation of vegetative bacteria, honey is known to harbor a variety of spore-forming bacteria [20,24]. It is therefore not surprising that we did not find spore killing activity even at 4× the MIC. We did, however, note that spore concentrations in as little as 1/4× MIC of honey remained near baseline levels over time in contrast to the steady increase in *C. difficile* spore concentrations observed in the absence of honey. Bactericidal activity was, likewise, not universal among our tested *C. difficile* isolates, in contrast to previous studies [12,13]. The results were dichotomous, with roughly half of isolates meeting general bactericidal criteria of MBC/MIC ≤ 4, and roughly half demonstrating MIC or MBC greater than the highest test concentration of 30% manuka honey. Others have noted that the microbiological definition of bactericidal is dependent on numerous factors and cannot alone predict outcomes in in vivo conditions [25]. The applicability of MIC and MBC values against *C. difficile* to achievable levels of honey in the colon is unknown at this time. 

Although we did not find Manuka honey to be universally bactericidal or inhibitory against *C. difficile* growth, its global effect on the gut microbiome and the host inflammatory response will be important additional considerations for potential use in CDI. Experimental models suggest a dose-dependent inhibition of *C. difficile* biofilm formation that is optimized at concentrations of 50% [26]. Manuka honey promotes healing by modulating the inflammatory response of the host and stimulating the proliferation of epithelial cells and fibroblasts [17], the properties which could aid in treating a damaged colonic epithelium, such as during CDI. Manuka honey has a more potent effect on Gram-positive than Gram-negative bacteria [27], which may be beneficial in restoring normal gut flora, which is preponderantly Gram-negative. Manuka honey is sold over-the-counter and is safe as a dietary supplement without allergenic effects or perturbation of the gut microbiome in studies of healthy volunteers [18]. Finally, manuka honey has been used animal models to successfully treat lower gastrointestinal infections without adverse effect [28]. There is also potential for use of manuka honey as an adjunct therapy to standard regimens of metronidazole or vancomycin. While resistance to vancomycin remains low overall for *C. difficile* (<5%), there are indications that resistance is increasing [5]. Synergistic activity between manuka honey and vancomycin has been observed for eradicating *Staphyloccoccus aureus* biofilm in vitro [29], but such studies are few and none have not been done for CDI.

## 4. Methods

### 4.1. Bacterial Isolates

*C. difficile* isolates consisted of non-toxigenic ATCC 70057, two previously characterized NAP1 strains (CDC, Atlanta, GA, USA), and 17 toxigenic clinical isolates including one typed as NAP1 by PCR. Clinical isolates were cultivated from diarrheal (Bristol scale 7) fecal specimens that tested positive for toxigenic *C. difficile* by *tcdB* PCR (GeneXpert, Cepheid, Sunnyvale, CA, USA) during routine clinical care of patients suspected to have CDI. Equal volumes of fecal specimen and 95% ethanol were vortexed for 10 s, then incubated at room temperature for 1 h. The suspension was plated on cycloserine cefoxitin fructose agar (Hardy Diagnostics, Santa Maria, CA, USA), then incubated at 35 °C for 48–72 h under anaerobic conditions. Colonies demonstrating characteristic yellow spreading colonies and a characteristic odor were definitively confirmed as *C. difficile* by mass spectrometry (Vitek MS, bioMeriuex, Durham, NC, USA).

### 4.2. Minimal Inhibitory and Bactericidal Concentration Determination

Minimum inhibitory concentrations for manuka honeys of four different MGO grades (MGO 30^+^, 100^+^, 250^+^, and 400^+^, Manuka Health, New Zealand) against the *C. difficile* isolates were determined by the broth microdilution method [30]. Stock solutions of 60% honey in BHI broth (Remel Inc., Lenexa, KS, USA) were sterilized by serial filtration through 0.45 µm and 0.22 µm polyvinylidene fluoride membranes (MilliporeSigma, Burlington, MA, USA) immediately prior to testing. *C. difficile* isolates were grown on CDC anaerobic blood agar for 48 h prior to MIC studies. A 2.0 McFarland suspension (~10^8^ colony-forming units (CFU)/mL) was prepared and inoculated 1:200 in the broth microdilution series (final concentrations 4, 6, 10, 14, 18, 22, 26, and 30% (*w*/*v*) honey) in a final volume of 100 µL. MICs were read after 48 h at 35 ± 2 °C anaerobic incubation. Growth and sterility controls were included for each organism tested and all MIC experiments were performed in duplicate. For MBC studies, baseline counts were obtained from the growth control well and 10 µL from each well showing no growth was plated to BHI agar. Plates were incubated anaerobically for 48 h at 35 ± 2 °C. Colony counts were taken and compared to baseline to determine MBC using a standardized CLSI method [31]. 

### 4.3. Sporidical Activity.

For sporicidal activity studies, we selected a *C. difficile* strain with an MIC of 6% and MBC of 30% in MGO 400+ honey to allow testing in honey concentrations up to 4× the MIC and to allow potential differences between viable cell counts and spore cell counts to be seen. A bacterial suspension of 2.0 McFarland was inoculated 1:10 in BHI broth in borosilicate tubes for the following conditions: ¼ ×, 1×, and 4× MIC, and no drug control, in a total volume of 7 mL each. Total viable and spore counts were performed at baseline and at 24, 48, 72, and 96 h after inoculation. Total viable cell count was performed by directly plating serial 10-fold dilutions of broth mixture to CDC anaerobic media in duplicate. Spore counts were performed by centrifuging 1 mL of broth mixture at 15,000× *g* for 2 min and resuspending the pellet in 1 mL of 50% ethanol. After 1-h incubation at room temperature, cells were again pelleted and washed twice in 1.0 mL phosphate buffered saline (PBS), then plated in serial 10-fold dilutions in duplicate on CDC anaerobic blood agar. All cell counts were taken after anaerobic incubation in 35 °C for 48 h.

### 4.4. Statistical Analysis

MIC results at the 50th percentile (MIC_50_) and the 90th percentile (MIC_90_) were analyzed by MGO grade. MIC values by MGO grade were further compared by the Kruskal–Wallis test (GraphPad Prism v8). Results were considered statistically significant if *p* < 0.05. Intra-assay reproducibility was calculated as the average coefficient of variation for each set of duplicate test results. For the purpose of statistical analysis, the higher MIC was used when 2 replicates yielded different MICs, and a value of 31% was used when the resulting MIC was >30%.

## 5. Conclusions

Using the broth microdilution method, we showed clinical isolates of *C. difficile* to be variably affected by manuka honey in terms of growth inhibition and bactericidal activity. The MGO grade of honey had no noticeable impact on overall MIC distributions or bactericidal activity. The correlations of the in vitro effects of manuka honey with the in vivo impact in CDI deserve to be explored further.

## Figures and Tables

**Table 1:** Summary statistics of minimal inhibitory concentrations and minimal bactericidal concentrations of different Manuka honey methylglyoxal (MGO) grades against 20 clinical isolates of toxigenic *C. difficile*.

Parameter	MGO30^+^	MGO100^+^	MGO250^+^	MGO400^+^
MIC Range	4 to >30%	4 to >30%	4 to >30%	6 to >30%
MIC_50_	14%	14%	10%	10%
MIC_90_	22%	22%	18%	14%
MBC = MIC, *n*	8	5	5	8
MBC = 1–2× MIC, *n*	2	2	3	1
MBC = 2–3× MIC, *n*	1	1	1	2
MBC >30%, *n*	9	12	11	9

## Supplementary Materials

The following are available online at https://www.mdpi.com/2079-6382/9/10/684/s1, Table S1: Raw MIC and MBC values for each isolate and manuka honey tested.
